# A Rare Coexistence: Achalasia Esophagus and Acute Intestinal Pseudo-Obstruction

**DOI:** 10.7759/cureus.62489

**Published:** 2024-06-16

**Authors:** Andreas Kyvetos, Anastasia Manoli, Panagiota Voukelatou, Theoni Theodoropoulou, Ioannis Vrettos

**Affiliations:** 1 2nd Department of Internal Medicine, General and Oncology Hospital of Kifissia “Agioi Anargyroi”, Athens, GRC; 2 2nd Department of Pediatrics, Pendelis General Children's Hospital, Athens, GRC

**Keywords:** clinica gastroenterology, interna medicine, gastrointestinal motility disorders, intestinal pseudo-obstruction, achalasia esophagus

## Abstract

Achalasia esophagus and acute intestinal pseudo-obstruction are distinct gastrointestinal motility disorders rarely found together in the same patient. We present a case of a 96-year-old woman exhibiting symptoms of both conditions, including dysphagia, regurgitation, abdominal distension, nausea, vomiting, and constipation. Diagnostic evaluations revealed esophageal dilation with a "bird beak" sign on timed barium swallows and significant bowel dilation without mechanical obstruction on computed tomography scans. Treatment involved conservative measures for acute intestinal pseudo-obstruction and palliative approaches for achalasia esophagus. The coexistence of these disorders raises questions about potential shared pathophysiological mechanisms involving the enteric nervous system or smooth muscle dysfunction. Further research is warranted to elucidate these connections and improve management strategies for such complex cases.

## Introduction

Achalasia is a rare esophageal motility disorder characterized by impaired relaxation of the lower esophageal sphincter (LES) and absent peristalsis, leading to symptoms such as dysphagia, regurgitation, and chest pain. Achalasia affects approximately 1.6 out of 100.000 individuals annually [1]. On the other hand, intestinal pseudo-obstruction manifests as bowel dilation without anatomical obstruction. Individuals exhibit symptoms akin to bowel obstruction, such as nausea, vomiting, abdominal swelling, and difficulty passing stool, often detectable through X-rays or CT scans. This condition can be acute (Ogilvie syndrome) or chronic [2].

The prevalence of acute intestinal pseudo-obstruction is higher among males and individuals aged 60 and above. It is estimated that approximately 100 cases per 100,000 inpatient admissions occur annually [3]. This condition is often diagnosed in hospitalized individuals post-surgery or following a significant illness [2].

The coexistence of these two conditions within the same patient is exceedingly rare, with only a limited number of cases reported in the medical literature. Herein, we present a case report detailing the diagnosis, management, and clinical outcomes of a patient diagnosed with both achalasia esophagus and acute intestinal pseudo-obstruction. Through this case, we aim to elucidate the challenges encountered in diagnosing and managing these concurrent gastrointestinal disorders, as well as explore potential underlying pathophysiological mechanisms linking the two conditions [4].

This article was previously presented as a meeting abstract at the 21st European Congress of Internal Medicine and the 12th International Congress of Internal Medicine, held in Athens, Greece, from March 15 to 18, 2023.

## Case presentation

A 96-year-old woman presented to the emergency department with persistent postprandial nausea and vomiting lasting over one month, along with a new onset of abdominal distension and constipation. Her medical history included anemia and arterial hypertension, and she was currently prescribed atenolol 50 mg once daily. Upon assessment, her vital signs were stable, with a pulse oximetry reading of 99%, blood pressure measuring 120/65 mmHg, and a body temperature of 36.6 °C. Cardiac auscultation revealed no murmurs, while respiratory auscultation revealed fine crackles in both lungs. The neurological examination yielded unremarkable findings. Notably, an abdominal examination indicated an absence of bowel sounds.

Laboratory investigations revealed mildly elevated levels of creatinine, urea, and inflammatory markers (Table 1).

**Table 1:** Blood test results WBC: white blood cell count; Hb: Hemoglobin; CRP: C-reactive protein; LDH: lactate dehydrogenase; AST: aspartate aminotransferase; ALT: alanine aminotransferase; K: potassium; Na: sodium; Ca: calcium

Parameter	Value	Normal range
Creatinine	1.4 mg/dl	0.6–1.4 mg/dl
Urea	100 mg/dl	10–50 mg/dl
WBC	6.44 K/μl	4.0–11.0 x 10^3^/μl
Hb	11.5 g/dl	12–16 g/dl
Platelets count	372 K/μl	150–450 K/μl
CRP	11.22 mg/dl	<0.5 mg/dl
Amylase	60 U/L	27–102 U/L
LDH	294 U/L	230–460 U/L
AST	20 U/L	9–36 U/L
ALT	15 U/L	10–28 U/L
K	4,1 mmol/L	3.5–5.5 mmol/L
Na	142 mmol/L	135–145 mmol/L
Ca	9.1 mg/dl	8.5–9.5 mg/dl

Further laboratory investigations revealed normal levels of vitamin B12, folate, and thyroid-stimulating hormone. Moreover, serological tests for the herpes simplex virus, cytomegalovirus, and Epstein-Barr virus were negative. Additionally, stool cultures and stool evaluations for toxin were negative for *Clostridioides difficile*.

**Figure 1:**
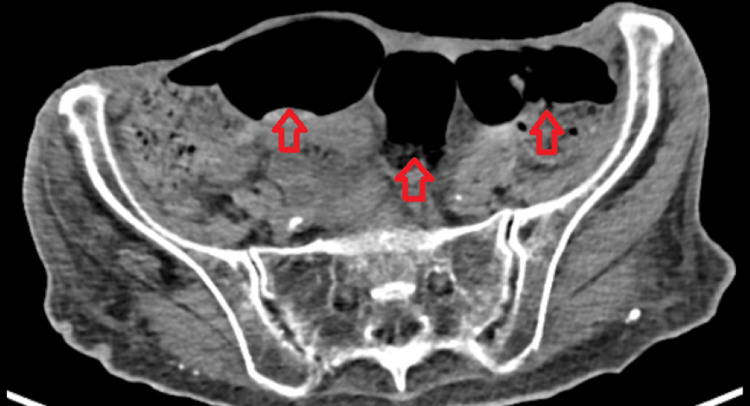
Abdominal CT scan Abdominal CT scan revealed largely dilated bowel loops (red arrows) with no signs of obstruction

**Figure 2:**
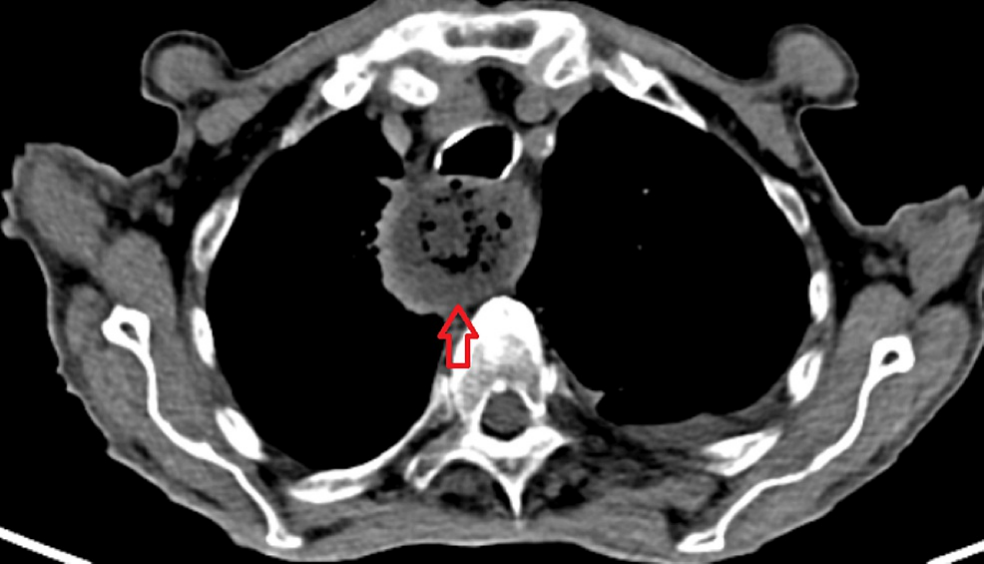
Thoracic CT scan Thoracic CT scan axial view revealed dilation of the thoracic esophagus (red arrow) with stenotic of the gastroesophageal junction

A full-body computed tomography (CT) scan demonstrated significant dilation of bowel loops without signs of obstruction, along with dilation of the distal thoracic esophagus and stenosis at the gastroesophageal junction (Figures 1-2). Additionally, the scan revealed condensed elements in both lungs, and the patient was prescribed moxifloxacin 400 mg once per day.

**Figure 3:**
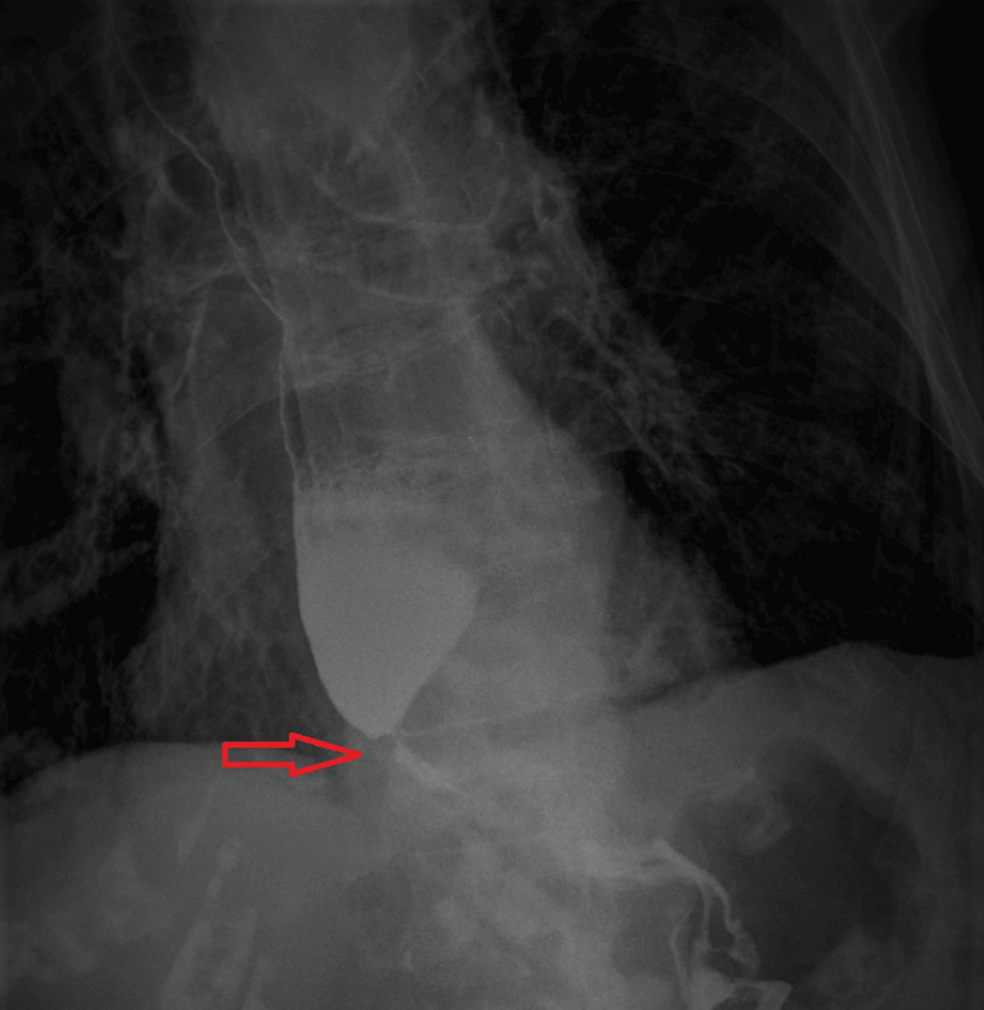
Timed barium esophagogram Timed barium esophagogram revealed esophageal dilation and narrowing of gastro-esophagus junction showing the classic ‘’bird beak’’ sign of the lower esophageal sphincter (red arrow)

Subsequent upper gastrointestinal tract endoscopy revealed esophageal dilation and substantial food retention, consistent with the findings from a timed barium esophagogram, which exhibited narrowing at the gastro-esophageal junction, displaying the classic "bird beak" sign indicative of lower esophageal sphincter dysfunction (Figure 3).

Therapeutically, the patient received metoclopramide, enemas, and nil per os, resulting in a remarkable improvement in symptoms. A follow-up CT scan confirmed a reduction in both bowel and esophageal dilation. Subsequently, the patient was referred to a gastroenterologist for further management.

## Discussion

The coexistence of an achalasia esophagus and acute intestinal pseudo-obstruction in a single patient presents a rare and challenging clinical scenario. Both conditions involve dysmotility of the gastrointestinal tract but affect distinct anatomical segments [1,2].

Diagnosing both achalasia esophagus and acute intestinal pseudo-obstruction requires a comprehensive evaluation encompassing clinical history, physical examination, and various diagnostic modalities. In our case, the patient presented with symptoms such as dysphagia, regurgitation, abdominal distension, nausea, vomiting, and constipation, raising a high index of suspicion for the presence of concurrent motility disorders.

Intestinal pseudo-obstruction CT imaging may reveal findings such as bowel dilation, air-fluid levels, and particularly notable proximal colonic dilatation extending from the cecum to the splenic flexure or even the rectum. It is imperative to rule out mechanical obstructions such as closed-loop obstructions, hernias, strictures, or masses during interpretation, such as in our case [2].

The possible reasons for the development of acute intestinal pseudo-obstruction encompass electrolyte imbalances, infections, and adverse reactions to medications, such as anticholinergics and opioids [2]. The patients in our case had no history of opioid use, diabetes, or any surgical procedures in the abdomen. Additionally, serologic testing for herpes simplex virus, cytomegalovirus, and Epstein-Barr virus was negative, as viral infections may cause inflammation of the enteric nervous system, causing intestinal pseudo-obstruction [2]. Patients with a dilated colon and rectum raise suspicion for toxic megacolon [2], but the stool evaluation for toxin was negative for Clostridioides difficile. Given the correlation between metabolic irregularities such as hypokalemia, hypomagnesemia, hypocalcemia, and intestinal pseudo-obstruction, a metabolic panel was conducted, and it was normal [2].

Achalasia esophagus diagnostic tests, such as esophagogastroduodenoscopy, esophageal manometry, and barium swallow studies, are essential for assessing esophageal and intestinal motility and identifying potential anatomical abnormalities. In our case, we performed a time-barium swallow study that revealed the "bird beak" sign of the lower esophageal sphincter, a characteristic finding of achalasia esophagus. Additionally, a gastrointestinal tract endoscopy revealed esophageal dilation and substantial food retention, consistent with the findings of the timed barium esophagogram. In a barium esophagogram, the typical presentation of the disease (subtype I) is characterized by a dilated, aperistaltic esophagus with a narrowed, tapering segment near the gastroesophageal junction, often described as resembling a "bird's beak" appearance [5].

Endoscopy is more likely to reveal the classic appearance in cases of moderate to severely dilated esophagus, while it may be less evident in early disease stages. Additionally, endoscopy serves a crucial role in ruling out pseudoachalasia or any mechanical obstruction that could present symptoms akin to achalasia [6]. In our case, the significant dilation observed during endoscopy strongly suggests that the onset of achalasia esophagus occurred a considerable time ago. The confirmation of achalasia diagnosis typically relies on high-resolution manometry (HRM), recognized as the current gold standard diagnostic test [6].

Therapeutically, the patient with acute intestinal pseudo-obstruction should undergo nil-per-os management while initiating nasogastric decompression. Conservative treatment typically involves nasogastric tube insertion for relieving proximal gut pressure, meticulous positioning adjustments, and sometimes rectal tube placement, occasionally preceded by limited tap water enemas. Although there are anecdotal accounts of success using conventional prokinetic agents like erythromycin, metoclopramide, and cisapride, their effectiveness appears sporadic, often yielding gradual improvements over a 12 to 24-hour period. Neostigmine stands out as the sole consistently effective option [7].

Achalasia, on the other hand, is a condition without a cure, and its precise cause remains uncertain [6]. The current therapeutic approaches for achalasia are primarily palliative, targeting the hypertonicity of the LES. The primary objectives of treatment are symptom alleviation, enhancement of esophageal emptying, and prevention of further esophageal dilation. Available modalities for managing achalasia encompass pharmacological, endoscopic, and surgical interventions [6].

According to the prevailing theory, achalasia arises from the selective depletion of inhibitory neurons in the myenteric plexus located in the distal esophagus and LES, resulting in an imbalance between excitatory and inhibitory signals. While excitatory neurons release acetylcholine, inhibitory neurons primarily release vasoactive intestinal peptides and nitric oxide [7]. The localized decrease in vasoactive intestinal peptide and nitric oxide levels, alongside increased excitatory activity, ultimately leads to malfunctioning of the LES and disruption of esophageal peristalsis [8,9].

On the other hand, acute intestinal pseudo-obstruction results from dysmotility of the gastrointestinal tract, typically involving the small and large intestines. The precise cause of acute intestinal pseudo-obstruction remains elusive. Nonetheless, evidence linking trauma, spinal anesthesia, and specific medications hints at a potential disruption of the autonomic nervous system [2]. The coexistence of intestinal pseudo-obstruction and achalasia is very rare, as only three cases exist in the medical literature [4]. In one of these cases, the patient first developed chronic intestinal pseudo-obstruction and then developed achalasia several years later. In the other two cases, such as in our case, the patient developed achalasia esophagus and subsequently acute intestinal pseudo-obstruction. Consequently, the two conditions, which rather share the same pathogenetic mechanism, can manifest in the same patient years apart, regardless of which one will manifest first.

## Conclusions

The coexistence of the achalasia esophagus and intestinal pseudo-obstruction suggests a potential commonality in their underlying pathophysiological mechanisms, possibly involving abnormalities in the enteric nervous system or smooth muscle dysfunction. However, further research is needed to elucidate the precise molecular and cellular pathways linking these two conditions. Until then, patients with one of these conditions should be monitored regularly to recognize the early signs of the other disease and take the necessary measures to relieve the patient's early symptoms before they become severe.
